# Osteoarthritis and nutrition. From nutraceuticals to functional foods: a systematic review of the scientific evidence

**DOI:** 10.1186/ar2016

**Published:** 2006-07-19

**Authors:** Laurent G Ameye, Winnie SS Chee

**Affiliations:** 1 Nutrition and Health Department, Nestlé Research Center, Vers-chez-les-Blanc, 1000 Lausanne 26, Switzerland

## Abstract

The scientific and medical community remains skeptical regarding the efficacy of nutrition for osteoarthritis despite their broad acceptation by patients. In this context, this paper systematically reviews human clinical trials evaluating the effects of nutritional compounds on osteoarthritis. We searched the Medline, Embase, and Biosis databases from their inception to September 2005 using the terms random, double-blind method, trial, study, placebo, and osteoarthritis. We selected all peer-reviewed articles reporting the results of randomised human clinical trials (RCTs) in osteoarthritis that investigated the effects of oral interventions based on natural molecules. Studies on glucosamine and chondroitin sulfate were excluded. The quality of the RCTs was assessed with an osteoarthritic-specific standardised set of 12 criteria and a validated instrument. A best-evidence synthesis was used to categorise the scientific evidence behind each nutritional compound as good, moderate, or limited. A summary of the most relevant *in vitro *and animal studies is used to shed light on the potential mechanisms of action. Inclusion criteria were met by 53 RCTs out of the 2,026 identified studies. Good evidence was found for avocado soybean unsaponifiables. Moderate evidence was found for methylsulfonylmethane and SKI306X, a cocktail of plant extracts. Limited evidence was found for the Chinese plant extract Duhuo Jisheng Wan, cetyl myristoleate, lipids from green-lipped mussels, and plant extracts from *Harpagophytum procumbens*. Overall, scientific evidence exists for some specific nutritional interventions to provide symptom relief to osteoarthritic patients. It remains to be investigated whether nutritional compounds can have structure-modifying effects.

## Introduction

Osteoarthritis (OA) is one of the most prevalent and disabling chronic diseases affecting the elderly. Its most prominent feature is the progressive destruction of articular cartilage which results in impaired joint motion, severe pain, and, ultimately, disability. Its high prevalence and its moderate-to-severe impact on daily life pose a significant public health problem [1].

Today, a cure for OA remains elusive. The management of OA is largely palliative, focusing on the alleviation of symptoms. Current recommendations for the management of OA include a combination of nonpharmacological interventions (weight loss, education programs, exercise, and so on) and pharmacological treatments (paracetamol, nonsteroidal anti-inflammatory drugs [NSAIDs], and so on) [2]. Among these pharmacological treatments, NSAIDs, despite serious adverse effects associated with their long-term use, remain among the most widely prescribed drugs for OA [3]. In this context, there is a need for safe and effective alternative treatments while the absence of any cure reinforces the importance of prevention.

Such prevention and alternative treatments could come from nutrition. It is now increasingly recognised that, beyond meeting basic nutritional needs, nutrition may play a beneficial role in some diseases [4]. OA as a chronic disease is the perfect paradigm of a pathology the treatment of which could be addressed by nutrition. By nature, nutrition is better positioned to provide long-term rather than short-term health benefits. This is because, in most cases, a nutritional compound has only limited effects on its biological target and relevant and significant differences are reached only over time through a build-up effect in which daily benefits add up day after day. For this reason, and because the time window for intervention is longer in chronic diseases, such diseases should, in theory, benefit more from nutrition than do acute diseases. In addition, because the mechanisms of cartilage degradation in OA are multifactorial and some nutritional compounds (such as plant extracts) usually contain multiple active compounds that target multiple pathways, nutrition could provide an alternative to pharmacological interventions whose often monomodal mode of action may explain their partial lack of clinical efficacy in OA. The attractiveness of using nutrition for OA also lies in the detriments that it can prevent. Long-term pharmacological interventions in OA are often associated with significant adverse effects. Nutraceuticals and functional foods could provide an advantageous alternative because, by regulatory laws, they have to be devoid of adverse effects.

There is no consensus on the definition of nutraceuticals and functional foods. The term 'nutraceutical' was coined from 'nutrition' and 'pharmaceutical' in 1989 by DeFelice and was originally defined as 'a food (or part of the food) that provides medical or health benefits, including the prevention and/or treatment of a disease' [5]. In a policy paper in 1999, Zeisel distinguished whole foods from the natural bioactive chemical compounds derived from them and available in a non-food matrix by using the term 'functional foods' to describe the former and nutraceuticals to describe the latter [6]. Under this newer definition (which we will use in the rest of this paper), nutraceuticals are thus functional ingredients sold as powders, pills, and other medicinal forms not generally associated with food. The term nutraceutical has no regulatory definition and is not recognised by the U.S. Food and Drug Administration, which uses instead the term 'dietary supplements' [7]. Some functional ingredients are sold as nutraceuticals in some countries but as drugs (that is, requiring medical prescription) in others. Compared with a nutraceutical/dietary supplement, a functional food is a food or drink product consumed as part of the daily diet [7,8]. It can be distinguished from a traditional food 'if it is satisfactorily demonstrated to affect beneficially one or more target functions in the body, beyond adequate nutritional effects in a way which is relevant to either the state of well-being and health or the reduction of the risk of a disease' [9]. A food product can be made functional by eliminating a deleterious ingredient, by adding a beneficial ingredient, by increasing the concentration of an ingredient known to have beneficial effects, or by increasing the bioavailability or stability of a beneficial ingredient [10]. In this paper, the beneficial ingredient supposed to provide the health benefit in a functional food or nutraceutical will be called functional ingredient. The functional ingredient in a functional food or in a nutraceutical/dietary supplement can be a macronutrient (for example, n-3 fatty acids), a micronutrient (for example, vitamins), or an ingredient with little or no nutritive value (for example, phytochemicals) [10].

In this context, the public interest in the benefits that nutrition could provide for OA is high. Numerous lay publications advertise the use of a whole range of nutraceuticals and functional foods for OA, and up to one out of five patients with OA uses such nonprescribed alternative medications [11], despite the fact that the mechanism of action of these products is often speculative and their efficacy not always supported by rigorous scientific studies. The aim of this paper was thus to review the available scientific evidence supporting the efficacy of the functional ingredients targeting OA and explaining their mechanism of action.

## Materials and methods

### Identification and selection of the literature

Systematic literature searches were performed to identify all human randomised clinical trials (RCTs) related to nutrition and OA. Computer databases used were Medline, Embase, and Biosis (searched from their respective inceptions to September 2005). Preliminary trial searches targeting specifically nutrition/nutraceuticals with lists of keywords such as 'food', 'supplements', 'plant', 'nutrition', 'vitamins', 'mineral', and 'nutraceuticals' performed poorly. Numerous valid trials that were already known to us were not selected by such searches. Hence, to be as exhaustive as possible, we changed our strategy and, instead of focusing on nutrition, devised a systematic search aiming at selecting all clinical trials in OA. This search of clinical trials in OA was fine-tuned for each database. Medline was searched by using the following strategy: random* AND (double-blind method [mh] OR (trial? OR stud??? OR placebo)) AND osteoarthritis [mh]. Embase was searched with the following keywords: (double near blind OR trial? OR stud??? OR placebo) AND osteoarthritis. Biosis was searched with the following keywords: random* AND (double near blind OR trial? OR stud??? OR placebo) AND osteoarthritis. These searches generated 1,519, 324, and 678 studies, respectively.

After the identical studies in the three searches were eliminated, the 2,026 remaining studies were individually screened based on their title and (if required) abstract or full content (Table 1). To be eligible for inclusion, a study had to fulfil all the following criteria: (a) to be a human RCT, (b) to investigate solely OA or (if investigating OA with other diseases) to report the results related to OA separately, (c) to be a peer-reviewed full paper (no restrictions on language), and (d) to investigate the effects of dietary/oral interventions focusing on natural molecules (as opposed to synthetic molecules). This last criterion is somewhat arbitrary. Its purpose was to separate the nutritional interventions from the pharmacological ones, a task which is far from trivial. Functional nutrition is a recent rapidly evolving field set at the border between foods and drugs, which explains why some ingredients, such as glucosamine, chondroitin sulphate, or S-adenosyl-L-methionine (SAMe), are registered as drugs in some countries but used in functional foods or as nutraceuticals in others. Because of this last criterion, studies focusing on SAMe were excluded from this review. Indeed, although a natural physiologic precursor of endogenous sulfated compounds, SAMe in its native form degrades rapidly and only stabilised synthetic forms have been used in scientific studies [12]. Studies dealing with glucosamine HCl, glucosamine sulphate, and chondroitin sulfate were excluded because several high-quality meta-analyses on these molecules have recently been published [13-16].

To look for further unidentified RCTs that met our inclusion criteria, a second search in PubMed was performed with OA and the name of each ingredient found through the primary search and also by screening the reference lists of all relevant articles identified. Finally, for all ingredients used in the RCTs selected that way, a systematic search limited to PubMed was performed to identify *in vitro *and animal studies related to this ingredient and articular cartilage. Among these studies, the most relevant ones were selected, and their results were reported to shed light upon the potential mechanisms of actions of these nutritional interventions.

### Quality assessment

This systematic review focuses on statistical differences in primary endpoints between treatment groups and considers the trials efficacious if the difference between groups was significant (*P *< 0.05) in placebo-controlled trials and not significant in NSAID-controlled trials. When no primary endpoint was mentioned, effects on visual analog scales (VASs), Lequesne functional index (LFI), and Western Ontario and McMaster universities (WOMAC) index were preferentially reported if available and used for the evaluation of efficacy.

The quality of each RCT related to a functional ingredient the efficacy of which was supported at least by one RCT was scored according to a standard set of 12 criteria based on published recommendations for the design of clinical trials in patients with OA [17-20] (Table 2). One point was assigned to each criterion that was met. If the criterion was not met or was not described at all, no point was assigned. The points were summed and divided by 12 in order to express the quality score as a percentage. A minus was placed in front of the score if the RCT did not support the efficacy of the intervention. Both authors scored the RCTs independently. Divergence was resolved by consensus after discussion. An RCT was considered of high quality when its OA-specific score was greater than or equal to 75%. Both authors also scored the RCTs with the validated Jadad score [21]. To determine and validate the robustness of our OA-specific score, the inter-individual variabilities of the two scores were calculated on the 42 graded RCTs. The inter-individual variabilities of the two scores were comparable and equaled 7% and 8%, respectively (that is, 7% to 8% of the individual criteria of the two scores end up with a different point between the two authors of this study).

### Best-evidence synthesis

A global score was then calculated to summarise the strength of evidence available for each functional ingredient (Table 3). To take into account the quality and quantity of RCTs, the global score was calculated by adding a factor to the mean quality score of the RCTs (that is, 0.33 when two positive high-quality RCTs were available, 0.66 when three positive high-quality RCTs were available, and 1.00 when four positive high-quality RCTs were available). Likewise, when two, three, or four negative high-quality RCTs were available, 0.33, 0.66, or 1, respectively, was subtracted from the mean quality score of the RCTs. Adding a factor gives more weight to the high-quality trials and helps to prevent the 'dilution' of the outcomes of high-quality trials when numerous low-quality trials exist. It also distinguishes the functional ingredients supported only by one, two, three, or four high-quality trials, which would otherwise end up with the same global score.

Consequently, the scores range from -2 to +2:

▪ A score below -0.5 corresponds to at least some evidence of inefficacy.

▪ A score between -0.5 and +0.5 indicates a lack of evidence of efficacy because it is obtained in case of conflicting evidence or when a majority of poor-quality trials are available.

▪ A score greater than 0.5 but less than or equal to 1 corresponds to limited evidence of efficacy because it is obtained when a majority of medium-quality trials exist in the presence of a maximum of one positive high-quality trial or when a single positive high-quality trial is available.

▪ A score between 1.01 and 1.33 indicates moderate evidence of efficacy because it requires two positive high-quality trials in the absence of major conflicting evidence.

▪ A score between 1.34 and 1.66 indicates good evidence of efficacy because it requires three positive high-quality trials in the absence of major conflicting evidence.

▪ A score between 1.67 and 2.00 indicates very good evidence of efficacy because it requires four positive high-quality trials in the absence of major conflicting evidence.

## Results

Out of the 2,026 identified studies, 52 RCTs that investigated the effects of functional ingredients in OA and that had their results reported in peer-reviewed full papers were identified. Historically, functional ingredients can be derived from primary food sources, from secondary food sources, from traditional medicinal products from all around the world, or from materials with no history of human exposure (for example, stanols from paper industry by-products for their cholesterol-lowering effects) [22]. The situation regarding OA is no different. Some ingredients included in this review are from primary food sources (for example, n-3 polyunsaturated fatty acids [n-3 PUFAs]), from secondary food sources (for example, ginger), from traditional medicinal products (for example, cat's claw), or from material with no history of human exposure as such (for example, 'hyperimmune' milk). The investigated nutritional interventions focused on lipids (avocado and soybean unsaponifiables [ASUs], n-3 PUFAs, lipid extracts from New Zealand green-lipped mussel, and cetyl myristoleate), on vitamins and minerals (vitamins C, E, B_3_, and B_12_, boron, a cocktail of vitamins and selenium, and a cocktail of minerals), on plant extracts (bromelain, *Rosa canina*, *Harpagophytum procumbens*, *Uncaria tomentosa*, and *Uncaria guianensis*, *Salix sp.*, ginger, turmerics, tipi tea, soy proteins, and *Boswellia serrata*), on a cocktail of plant extracts (SKI306X, Gitadyl, Duhua Jushing Wan, and Articulin-F), and on a few other types of ingredients (methylsulfonlymethane, hyperimmune milk, and collagen hydrolysate).

### Lipids

#### Avocado/soybean unsaponifiables

The most thoroughly investigated lipid mixture is Piascledine (Pharmascience, Inc., Montreal, Quebec, Canada). Piascledine is composed of one third avocado and two thirds soybean unsaponifiables (ASUs), the oily fractions that, after hydrolysis, do not produce soap [23].

Four double-blind placebo-controlled RCTs (Table 4) and one systematic review evaluated ASUs on knee and hip OA [24-28]. In two 3-month RCTs, one on knee and hip OA [24] and one solely on knee OA [25], 300 mg once a day decreased NSAID intake. No statistical difference in any primary or secondary endpoints was detected between 300 and 600 mg once a day [25]. In a 6-month RCT on knee and hip OA, 300 mg once a day resulted in an improved LFI compared with placebo [26]. ASUs had a 2-month delayed onset of action as well as residual symptomatic effects 2 months after the end of treatment. In a 2-year RCT on hip OA, 300 mg once a day did not slow down narrowing of joint space width [27]. In addition, none of the secondary endpoints (LFI, VAS of pain, NSAID intake, and patients' and investigators' global assessments) was statistically different from placebo after 1 year. However, a post hoc analysis suggested that ASUs might decrease narrowing of joint space width in patients with the most severe hip OA. In summary, although ASUs might display medium-term (several months') symptom-modifying effects on knee and hip OA, their symptom-modifying effects in the long term (>1 year) have not been confirmed. ASUs might slow down narrowing of joint space width in patients with severe hip OA, but this requires confirmation. Based on our best-evidence synthesis, good evidence is provided by ASUs for symptom-modifying effects in knee and hip OA but at the same time, there is some evidence of absence of structure-modifying effects (Table 3). A recent systematic review on ASUs recommended further investigation because three of the four rigorous RCTs suggest that ASUs is an effective symptomatic treatment, but the long-term study is largely negative [28]. However, the fact that this long-term study was primarily aiming at demonstrating structure-modifying and not symptom-modifying effects might explain why no symptomatic effects from ASUs were detected in the long-term study. Indeed, symptoms and structural damage are known to mildly correlate in OA, and the most appropriate patients to demonstrate a structure-modifying effect might not be the most appropriate to demonstrate a symptom-modifying effect. As for safety, none of the four RCTs reported significant differences in adverse effects between ASUs and placebo.

In sheep with lateral meniscectomy, 900 mg once a day for 6 months reduced the loss of toluidine blue stain in cartilage and prevented subchondral sclerosis in the inner zone of the lateral tibial plateau but not focal cartilage lesions [29].

*In vitro*, ASUs display anabolic, anticatabolic, and anti-inflammatory effects on chondrocytes. ASUs increased collagen synthesis [30] and inhibited the spontaneous and interleukin (IL)-1β-induced collagenase activity [23,31]. They increased the basal synthesis of aggrecan and reversed the IL1β-induced reduction in aggrecan synthesis [32]. ASUs were also shown to reduce the spontaneous and IL1β-induced production of matrix metalloproteinase (MMP)-3, IL-6, IL-8, and prostaglandin E2 (PGE2) while weakly reversing the IL1β-induced decrease in TIMP (tissue inhibiting metalloproteinase)-1 production [23,30,32]. One study showed that ASUs decreased the spontaneous production of nitric oxide (NO) and macrophage inflammatory protein-1β [32] while stimulating the expression of transforming growth factor-β and plasminogen activator inhibitor-1 [33]. This stimulated production of plasminogen activator inhibitor-1 could decrease MMP activation.

The effects of avocado unsaponifiables alone, of soybean unsaponifiables alone, and of three mixtures of ASUs, were compared [23,32]. The mixtures were A1S2 (Piascledine), A2S1, and A1S1, with respective ratios of ASUs of 1:2, 2:1, and 1:1. All mixtures significantly reduced the spontaneous production of IL-6, IL-8, and PGE2 and the IL1β-induced production of PGE2. A1S2 and A1S1, but not A2S1, significantly reduced the spontaneous and IL1β-induced production of MMP-3 and the IL1β-induced increase in collagenase activity, but only A1S2 inhibited the spontaneous collagenase activity. For some parameters, avocado unsaponifiables or soybean unsaponifiables alone were as potent as mixtures. In some cases, a single source of unsaponifiables seemed to be active. In other cases, both sources of unsaponifiables were active with synergistic or counteracting effects. The superiority of Piascledine over different ASU mixtures or over avocado or soybean unsaponifiables alone thus remains to be demonstrated.

#### Omega-3 PUFAs

PUFAs are classified as n-3, n-6, or n-9 depending on the position of the last double bond along the fatty acid chain. In n-3, this last double bond is located between the third and fourth carbon atom from the methyl end of the fatty acid chain. The main dietary PUFAs are n-3 (such as linolenic acid and eicosapentenoic acid) and n-6 (such as linoleic acid and arachidonic acid). Omega-3 is found in soybean and canola oils, flaxseeds, walnuts, and fish oils, whereas n-6 is found in safflower, corn, soybean, and sunflower oils as well as in meat. The modern Western diet is relatively low in n-3 PUFAs and relatively high in n-6 compared with the diet in Western pre-industrialised societies or with the modern Eastern diet. The n-6/n-3 ratio is 25:1 in the modern Western diet compared with 2:1 in Western pre-industrialised societies. A high n-3 intake correlates with a low incidence of cardiovascular and inflammatory diseases [34,35]. The utility of n-3 for OA remains to be shown. In a 24-week double-blind placebo-controlled RCT, 10 ml of cod liver oil per day containing 786 mg of eicosapentaenoic acid, in addition to treatment with NSAIDs, did not decrease the VAS of pain or disability [36].

The articular cartilage content of arachidonic acid, a n-6 precursor of the pro-inflammatory eicosanoid PGE2, correlates with OA severity [37]. n-3 and n-6 are metabolised by cyclo-oxygenases (COXs) and lipo-oxygenases (LOXs) into distinct eicosanoids. The n-6-derived eicosanoids tend to be pro-inflammatory, whereas the n-3-derived eicosanoids tend to be anti-inflammatory. Hence, a high proportion of n-3 is supposed to lead to a relative deficiency in pro-inflammatory n-6 metabolites [34]. Dietary lipid interventions in animals modified the PUFA composition of articular cartilage [38], suggesting that high n-3 intake could have a beneficial effect on cartilage metabolism. In addition to eicosanoids, the anti-inflammatory effect of n-3 could also be mediated by their newly discovered oxygenated derivatives called resolvins, which through their binding to G protein-coupled receptors act as potent antagonists of inflammation [39].

The *in vitro *effects of 10 to 100 μg/ml of n-3 (linolenic, eicosapentaenoic, and docosahexaenoic acids) on chondrocytes have been investigated [40-42]. n-3 did not affect the spontaneous or the IL1-induced decrease in glycosaminoglycan (GAG) synthesis, but dose-dependently inhibited the IL1-induced GAG degradation. n-3 dose-dependently decreased the IL1-induced aggrecanase activity and basal aggrecanase and collagenase activity, whereas, in contrast, n-6 stimulated the basal aggrecanase and collagenase activity. n-3 also decreased the IL1-induced mRNA expression of ADAMTS-4 (aggrecanase), COX-2, 5-LOX, FLAP (5-LOX-activating protein), IL1α, and tumour necrosis factor (TNF) α and the basal mRNA levels of these genes. Finally, n-3 decreased the basal and IL1β-induced mRNA and protein levels of MMP-3 and MMP-13. All these parameters were unaffected by n-6 PUFAs. Taken together, these results indicate that n-3 PUFAs have anticatabolic and anti-inflammatory properties. Nevertheless, too low of an n-6/n-3 ratio can be detrimental. A diet with very low levels of n-6 PUFAs induced occasional surface irregularities and localised proteoglycan depletion in cartilages in rats [38].

#### Lipid extract from New Zealand green-lipped mussel (*Perna canaliculus*)

The incidence of arthritis in coastal-dwelling Maoris is low, possibly due to their high consumption of green-lipped mussels. The powder and lipid extracts from this mussel have been investigated in OA (Table 4). These products contain n-3 PUFAs as well as vitamins associated or not associated with chondroitin sulfate, amino acids, and minerals [43,44]. In a 6-month double-blind placebo-controlled RCT on knee OA, Seatone™ (McFarlane Laboratories, Auckland, New Zeland), a mussel gonad extract, improved four endpoints (VAS of pain, functional index, and patients' and physicians' overall assessments) out of 10 investigated but only in patients with mild to moderate OA [45]. In a 3-month double-blind RCT, 350 mg of Seatone™ three times a day improved VAS of pain in 40% of patients versus 13% in placebo [46]. In a small 3-month double-blind RCT, 1,150 mg/day of a mussel powder and 210 mg/day of a lipid extract decreased the values of a VAS of pain [47]. However, the small number of enrolled patients and the absence of placebo group, complete blinding, and baseline characteristics of the population seriously limit the relevance of this trial. No serious adverse effects have been reported. According to the best-evidence synthesis (Table 3), there is limited evidence of efficacy for green-lipped mussel in OA. This is in agreement with a recent systematic review evaluating the effectiveness of this ingredient for OA and rheumatoid arthritis [48].

In a 6-week double-blind placebo-controlled RCT in dogs, 1 g per day of a green-lipped mussel powder sprinkled on the food or on semi-moist treats or directly incorporated as 0.3% in a dry diet significantly improved total arthritic score, joint pain, and joint swelling [44,49]. More than 50% of the dogs demonstrated a 30% or greater reduction in total arthritic score. However, it is unclear whether these dogs suffered from OA specifically, and the disease severity was not described.

#### Cetyl myristoleate

The oil cetyl myristoleate is the hexadecyl ester of the unsaturated fatty acid cis-9-tetradecenoic acid, commonly named myristoleic acid. Whereas myristoleic acid is commonly found in fish oils, whale oils, and dairy butter, cetyl myristoleate is known to exist only naturally in sperm whale oil and in a small gland in the male beaver. It can be synthesised by esterification of myristoleic acid. Although cetyl myristoleate is claimed to be beneficial for OA, there is lack of scientific evidence to support its efficacy. Nevertheless, a 68-day placebo-controlled single-blind RCT on severe knee OA (that is, LFI >14) concluded that three 500 mg capsules, containing 350 mg of a blend of olive oil and various cetylated fatty acids, 50 mg of lecithin, and 75 mg of fish oil, twice a day, significantly increased knee flexion compared with placebo [50] (Table 4). According to the best-evidence synthesis (Table 3), this low-quality RCT provides limited scientific evidence of efficacy for cetyl myristoleate. Hence, further research is needed to evaluate the safety and potential benefits of cetyl myristoleate and cetylated fatty acids in the treatment of OA.

### Vitamins and minerals

Due to their antioxidant properties, vitamins could have beneficial effects in OA [51,52]. Usually, antioxidant defences neutralise most reactive oxygen species (ROS) by enzymes such as superoxide dismutase, catalase, and peroxidase or by small antioxidant molecules. However, when ROS are produced in increased amounts like in OA, the antioxidant capacity of cells and tissues can become insufficient to detoxify the ROS, which then contribute to cartilage degradation by inhibiting matrix synthesis, directly degrading matrix molecules, or activating MMPs (reviewed in [53]).

The effects of vitamins C, E, and B on OA have been formally investigated in RCTs (see below). One obvious candidate not yet evaluated is vitamin D (vit D). Pathophysiological changes in OA affect periarticular bone, and normal bone metabolism requires vit D. Hence, suboptimal levels of vit D may impair bone metabolism and predispose to OA. In addition, the expression of vit D receptors is upregulated in human OA chondrocytes [54]. The Framingham study found a threefold increase in risk of OA progression for patients in the middle and lowest tertiles of serum levels of 25-vit D [55]. Low serum levels of vit D also predicted loss of joint space and osteophyte growth. The Study of Osteoporotic Fractures in women found that the risk of incident hip OA defined by joint space narrowing was increased for patients in the middle and lowest tertiles of serum levels of 25-vit D_3 _[56]. Based on these data, an RCT testing the efficacy of vit D may be required.

#### Vitamin C

Vitamin C (vit C) is a common term used for L-ascorbic acid, dehydro-L-ascorbic acid (the oxidised from of L-ascorbic acid), and L-ascorbic acid salts (sodium, potassium, and calcium L-ascorbate). L-ascorbic acid constitutes the majority (80%-90%) of vit C in food. It is found in rose hips, blackcurrants, and citrus fruits but can also be synthesised from glucose.

The Framingham epidemiological study found a threefold reduction in risk of OA progression for both the middle and highest tertiles of vit C intake and an inverse association between vit C intake and cartilage loss [57]. No association was found between vit C intake and osteophyte growth or rate of apparition of the disease. In this study, vit C intake was assessed using a food frequency questionnaire, which can induce errors and bias. In a 14-day double-blind crossover RCT on knee and hip OA, 1 g twice a day of calcium ascorbate was more efficient than placebo in decreasing VAS of pain [58] (Table 4). Although the quality of the RCT was high (Table 3), the measured effect was small (a 4.6-mm decrease from a starting basal level of 50 mm) and obtained with a dose equal to the upper tolerable intake level for adults (that is, the highest level of daily intake from food, water, and supplements which is likely to pose no risk of adverse health effects for almost all individuals in the general population), well above the current recommended daily allowances (RDAs) of 60 to 200 mg per day. The long-term safety of such high doses of vit C in elderly patients with OA needs to be evaluated, and efficacy needs to be confirmed by longer RCTs.

Guinea pigs, like humans, possess a nonfunctional gene for L-gulono-γ-lactone oxidase, which makes them unable to synthesise ascorbic acid and dependent on dietary ascorbic acid to prevent scurvy. In guinea pigs, a 'megadose' of 150 mg per day of ascorbic acid decreased the severity of surgically induced knee OA [59,60] but increased severity of spontaneous OA [61], despite the ability of ascorbic acid to increase cartilage collagen content. A third guinea pig trial stated, on the contrary, without providing any details, that a fivefold increase of ascorbic acid to the drinking water (equivalent to 1 g per liter) had a slight chondroprotective effect on the development of spontaneous lesions but not on surgically induced OA [62]. In view of these conflicting results and in the absence of strong evidence of efficacy in humans, it was recommended that vit C intakes for OA not exceed the current RDA [61].

Articular cartilage accumulates ascorbic acid [63]. In chondrocytes, ascorbic acid and dehydroascorbate are transported, respectively, through the sodium-dependent vit C transporter (SVCT)-2 [64] and the glucose transporter GLUT 1 [65]. In OA, the majority of vit C is expected to be transported through SVCT-2 [65]. Ascorbic acid serves as a cofactor for prolyl and lysyl hydroxylases, enzymes crucial in collagen synthesis. *In vitro*, ascorbate and ascorbic acid increased protein and proteoglycan synthesis by articular chondrocytes [64,66,67] and increased the mRNA levels of type I and II collagen [64,68] and aggrecan and α-prolyl 4-hydroxylase [64]. It decreased the lipopolysaccharide (LPS)-induced GAG release [69]. It also affected the activities of lysosomal enzymes, decreasing the activities of arylsulfatase A and arysulfatase B, an *N*-acetylgalactosaminidase-4-sulfatase, but increasing the activity of acid phosphatase in normal and OA chondrocytes [66].

Ascorbic acid can cross-link collagen and other proteins by non-enzymatic glycation, leading to the formation of advanced glycation endproducts (AGEs). Threose, a metabolite of ascorbic acid, increases the AGE content of articular cartilage *in vitro *[70]. These cross-links increase the stiffness of the collagen network, which is hypothesised to increase cartilage susceptibility to OA. High *in vitro *levels of ascorbic acid (756 μM) also increased protein carbonylation, one type of oxidative damage [64]. However, when guinea pigs were fed with diets containing different levels of ascorbic acid, no changes in the AGE content of articular cartilage were detected [61].

#### Vitamin E

Natural vitamin E (vit E) comprises eight different forms, α-, β-, γ-, and δ-tocopherol and α-, β-, γ-, and δ-tocotrienol, produced solely by plants. One of the richest food sources of vit E is edible plant oils. Synthetic α-tocopherols (the eight other possible side-chain stereoisomers besides the natural one) and their esters (α-tocopherylsuccinate and α-tocopherylacetate) also exist. α-Tocopherylacetate is often used commercially because vit E esterification protects it from oxidation. In the human body, the ester is rapidly cleaved by cellular esterases making natural vit E available.

Five RCTs have tested the natural form of vit E or α-tocopherylacetate. Two trials concluded that vit E was more efficient than placebo in decreasing pain. In a small 10-day single-blind crossover RCT on mainly spondylosis, 600 mg of vit E per day was superior to placebo as assessed by a patient questionnaire [71], whereas in a 6-week double-blind RCT on OA, 400 IU of α-tocopherylacetate once a day was superior to placebo as assessed by a joined patients' and doctors' global assessment of pain [72]. One trial suggested that vit E was no less efficient than diclofenac in decreasing pain. In a 3-week double-blind RCT on OA, no significant difference was found between 544 mg of α-tocopherylacetate three times a day and 50 mg diclofenac three times a day on VAS of pain [73]. However, the two most recent trials failed to show any benefit over placebo on knee OA. In a 6-month double-blind RCT, 500 IU of vit E a day showed no symptomatic benefit over placebo as assessed by WOMAC [74], whereas in a 2-year double-blind RCT, 500 IU of vit E a day showed no symptomatic or structure-modifying benefit over placebo as assessed by magnetic resonance imaging or WOMAC, respectively [75]. Although three out of the five RCTs concluded that vit E decreased pain, the two longest, largest, and highest-quality trials (Table 3) failed to detect any symptomatic or structural effects in knee OA. This suggested that, at least for knee OA, vit E alone has no medium-term beneficial effect. According to the best-evidence synthesis (Table 3), there is no evidence of symptom-modifying efficacy for vit E and some evidence of inefficacy regarding structure-modifying effects. No significant adverse event was reported.

Only a few papers investigated the *in vitro *effects of vit E on chondrocytes. Tiku *et al*. [76] showed that when chondrocytes were submitted to an oxidative burst, vit E reduced the catabolism of collagen by preventing the protein oxidation mediated by aldehydic downproducts of lipid peroxidation. Vit E strongly increased the sulfate incorporation while slightly reducing the glucosamine incorporation [77], suggesting that it increased GAG sulfatation or that it increased GAG synthesis while reducing glycoproteins or glycolipids synthesis. Like vit C, vit E affected the activities of lysosomal enzymes: it decreased the activities of arylsulfatase A and of acid phosphatase in cultures of human articular chondrocytes [77]. However, vit E did not affect the LPS-induced catabolism of GAGs [69] and did not prevent synoviocyte apoptosis induced by superoxide anions [78].

#### Vitamins B

In a 3-month double-blind RCT of high quality (Table 3), a megadose of niacinamide (vitamin B_3_, 500 mg six times per day) was more efficient than placebo in reducing drug intake and symptoms but not pain [79] (Table 4). Such a high dose is 2 orders of magnitude above the upper tolerable intake level and is of concern [80].

A 2-month double-blind crossover RCT in hand OA comparing 6,400 μg of folate with or without 20 μg of cobalamin (vitamin B_12_) daily, to placebo, had no significant effects on mean hand grip values [81].

#### Cocktail of vitamins and selenium

Two cocktails of vitamins with added selenium, a component of the antioxidative enzyme glutathione peroxidase, have been investigated. In a small pilot 6-month double-blind placebo-controlled RCT, a mixture of vitamins A, C, and E (in undisclosed amounts) and 144 μg of selenium per day had no effect on VAS of pain or stiffness [82]. Vitamins A, C, E, B2, and B6 and selenium decreased OA incidence and severity in STR/1N mice, possibly through an antioxidant effect because the expression of two antioxidative enzymes, glutathione peroxidase and superoxide dismutase, was increased in cartilage [83].

#### Boron

Femoral OA bone contains less boron, a nonmetallic trivalent chemical element, than does normal bone [84], suggesting that boron might have a beneficial effect in OA. A small 8-week double-blind placebo-controlled RCT suggested that 6 mg/day, taken as sodium tetraborate decahydrate, was more efficient than placebo in reducing a patients' assessment scale of symptoms [85] (Table 4). This RCT, however, is of low quality (Table 3); hence, longer and higher-quality RCTs are required to evaluate thoroughly the benefits of boron for OA.

#### Cocktail of minerals

Sierrasil, a cocktail of 36 minerals from the Sierra Mountains in the U.S., was tested at two doses (2 or 3 g/day) and at a dose of 2 g/day with a cat's claw extract (100 mg/day) in an 8-week double-blind placebo-controlled RCT on knee OA [86]. None of these treatments was more effective at relieving symptoms than placebo as assessed by WOMAC or VAS of pain.

### Phytochemicals and plant extracts

#### Bromelain

Bromelain is a crude, aqueous extract obtained from both the stems and immature fruits of the pineapple plant (*Ananas comosus *Merr, mainly var Cayenne from the family of bromeliaceae), which contains a number of proteolytic enzymes. Bromelain was suggested to have anti-inflammatory, analgesic, antioedematous, antithrombic, and fibrinolytic effects, although many of the studies describing these properties were of poor quality ([87], and reviewed in [88]). Three different preparations containing bromelain mixed with diverse enzymes have been tested in nine trials on knee OA ([87,89,90] for a review and references therein). Bromelain was taken in tablets coated to resist stomach digestion at a daily dose ranging from 270 to 1,890 mg. All trials might have been insufficiently powered, were of short duration (3 to 6 weeks), and enrolled OA patients with flare-up episodes. Most used the LFI as a primary endpoint. Although bromelain was as effective or more effective effective than 100 to 150 mg of diclofenac per day in seven trials, it was also not more efficient than placebo in two other trials. This absence of efficacy over placebo, coupled with the fact that in some diclofenac-controlled trials the LFI or other functional endpoints continued to decrease in both groups even 4 weeks after the end of treatment [91,92], suggested a possible spontaneous resolution of the flare-up episode rather than a real efficacy of the treatment. Longer trials of higher quality were advocated to confirm the efficacy of bromelain [87]. The best-evidence synthesis indicates a limited evidence of efficacy based on five trials (Tables 3 and 4). Lack of sufficiently detailed data prevented the inclusion of the four other trials.

In one trial, a high dose of bromelain (945 mg/day) induced a higher incidence of adverse effects and dropouts compared with diclofenac [92], whereas in another trial, a lower dose (270 mg/day) induced a higher dropout rate due to adverse effects than diclofenac [90]. Together, these two reports question the safety and tolerability of bromelain.

#### *Rosa canina*

A standardised rose-hip powder made from the seeds and husks of the fruits from a subtype of *R. canina *(Hyben Vital™ produced by Hyben Vital International, Langeland, Denmark), the common wild-briar rose of English hedgerows, was evaluated in three RCTs. In a 4-month double-blind RCT on hip and knee OA, 2,500 mg of this powder twice a day did not improve active or passive mobility (joint rotation, flexion, and extension) more than placebo, except for passive hip flexion [93]. In a 3-month crossover double-blind RCT, 2,500 mg of *Rosa *powder twice a day decreased pain (as measured by a categorical scale) more efficiently than placebo when placebo was given first but not when it was given second [94]. No pain difference was found when the two groups were compared before crossover either. This RCT, which enrolled patients with OA in various joints, did not include any washout period. In a 3-month crossover double-blind RCT on knee and hip OA which included a 3-week washout period, 2,500 mg of *Rosa *powder twice a day was more effective than placebo in decreasing WOMAC pain after 3 weeks of treatment but not after 3 months [95]. The lack of significance after 3 months could have been due to the decreased paracetamol consumption observed when patients were under active treatment. At 3 months, the *Rosa *treatment decreased the WOMAC function and stiffness subscales (secondary endpoints) more efficiently than placebo. According to the best-evidence synthesis, there is a lack of scientific evidence for *R. canina *extracts. However, the last RCT suggests that *R. canina *powder might have some efficacy. Hence, further research on this extract is required before making any conclusion about its efficacy or lack of efficacy. No major side effects were reported in these three RCTs.

Daily intake of 45 g of rose hip powder reduced chemotaxis of peripheral blood neutrophils and serum creatinine and C-reactive protein (CRP) levels in healthy and OA subjects [96,97].

#### *Harpagophytum procumbens *(devil's claw)

*H. procumbens*, also called devil's claw, is a South African plant that grows in regions bordering the Kalahari. Secondary tuberous roots are used to prepare powders or extracts, which were tested in several RCTs (reviewed in [98]). Product standardisation is based on the content of harpagoside, the principal compound found in the raw material. In all RCTs, the harpagoside content was similar and greater than 50 mg/day.

In a 2-month double-blind RCT on spine and knee OA, 670 mg of powder three times a day was more efficient than placebo in reducing VAS of pain [99] (Table 4). In a 4-month double-blind diacerhein-controlled RCT on hip and knee OA with flare-up episodes at inclusion, 2.6 g of powder/day was no less efficient than 200 mg of diacerhein per day in improving VAS of pain [100,101]. Devil's claw was also better tolerated than diacerhein. A systematic review of the efficacy of *Harpagophytum *for OA concluded that there is limited evidence of efficacy for ethanolic extract when providing less than 30 mg of harpagoside per day in the treatment of knee and hip OA and moderate evidence of efficacy for the use of powder when providing 60 mg of harpagoside daily in the treatment of spine, hip, and knee OA [102]. The best-evidence synthesis used here (Table 3) indicates a limited evidence of efficacy. Whether the efficacy differs between patients with flare-up episodes or without is currently not clear. The need for larger, better designed RCTs with higher doses has been advocated before making categorical recommendations for *Harpagophytum *[98]. No safety concerns appeared from the 4,300 patients, who received *Harpagophytum *products. In an uncontrolled surveillance study, 0.8 g of an aqueous extract three times a day reduced blood sedimentation time and CRP levels [103]. An extract reduced the IL1β-induced production of MMP-1, MMP-3, and MMP-9 proteins by chondrocytes [104].

#### *Uncaria tomentosa *and *Uncaria guianensis *(cat's claw)

Cat's claw is a vine from the basin of the Amazon River. There are two species, *U. tomentosa *and *U. guianensis*, that are traditionally used in South America for their anti-inflammatory properties. The bark and the root are prepared as an extract in hot water. Product standardisation is based on alkaloid content, although *U. guianensis *extracts are more potent than *U. tomentosa *extracts *in vitro *despite a much lower alkaloid content [105]. Because numerous HPLC (high-performance liquid chromatography) fractions are biologically active *in vitro*, *in vivo *efficacy might be due to multiple compounds.

In a small 4-week double-blind RCT on knee OA, 100 mg of an extract from *U. guianensis *once a day was more efficient than placebo in reducing pain associated with activity but not pain at rest or at night [106] (Table 4). There was no statistical difference between groups in reported adverse effects in this trial, although cat's claw has been reported as nephrotoxic [107]. According to the best-evidence synthesis (Table 3), this low-quality RCT does not provide scientific evidence of efficacy for cat's claw. If cat's claw is proven efficacious in the future, a carefully controlled process of production will probably be required to prevent any nephrotoxicity.

#### *Salix sp. *(willow bark)

The anti-inflammatory, antipyretic, and analgesic effects of willow bark have been known since antiquity. Willow bark contains salicin, which is rapidly metabolised into salicylic acid. The acetylated derivative of salicylic acid is known as aspirin. Willow bark extracts are usually standardised based on salicin content even if salicin might not be the major active compound.

In a 2-week double-blind RCT on knee and hip OA, an amount of extract corresponding to 240 mg of salicin a day was more efficient than placebo in reducing WOMAC pain subscore, but the effect was small [108]. Another 6-week double-blind RCT comparing the effects of another willow bark extract at the same dose with placebo and diclofenac 50 mg twice a day on knee and hip OA confirmed the efficacy of diclofenac but failed to detect any significant difference between willow bark and placebo on WOMAC pain subscore [109]. In another 2-month RCT, a cocktail of five plants, which included 100 mg of willow bark, failed to conclusively demonstrate an analgesic effect [110]. Skin allergic reactions have been linked to *Salix *ingestion in 3% to 11% of patients [111]. The best-evidence synthesis (Table 3) indicates a lack of evidence of efficacy for *Salix *extracts.

#### Ginger and turmeric

The *Zingiberaceae *family includes gingers and turmerics. Ginger is a very popular spice with a world production of 100,000 tons a year. It is used in traditional Japanese Kampo, Ayurvedic, and Chinese medicine as an anti-inflammatory agent for musculoskeletal diseases. Three RCTs evaluated ginger extracts prepared from the rhizomes of *Zingiber officinale *and *Alpinia galanga*.

In a double-blind RCT on knee OA, after crossover at 6 months, but not at 3 months before crossover, 250 mg of an extract of *Z. officinale *four times a day reduced VAS of pain and handicap more efficiently than placebo [112]. In a 6-week double-blind RCT on knee OA, 255 mg of an extract of *Z. officinale *and *A. galanga *twice a day was more efficient than placebo in reducing knee pain on standing up based on the percentage of responders [113]. The RCT suffered from incomplete blinding, and the beneficial effects were small and not observed on WOMAC and quality of life [114]. Finally, a 3-month three-way crossover double-blind RCT compared the efficacy of another ginger extract with ibuprofen and placebo in knee and hip OA but failed to demonstrate a difference between placebo and ginger extract as assessed by VAS of pain and LFI [115]. The best-evidence synthesis (Table 3) indicates a lack of evidence of efficacy. In two RCTs, a higher number of adverse effects and higher dropout rates related to adverse effects in ginger groups [112,113] question the safety of these extracts.

*In vitro*, ginger extract decreased the IL1β- and LPS-induced production of NO and PGE2 by OA cartilage [116]. In synoviocytes, it decreased the IL1β- or TNF-α-induced expression of TNF-α mRNA and protein, the TNF-α-induced production of COX2, and the TNF-α-induced activation of nuclear factor (NF)-κB by reducing the protein level of the NF-κB inhibitor IκB [117].

An extract prepared from the Indian and Javanese turmerics *Curcuma domestica *and *Curcuma xanthorriza*, was tested on hip and elbow OA in an 8-week double-blind randomised trial in dogs [118]. No significant difference on the kinetic gait analysis was found between extract and placebo.

#### Flavonoids

Flavonoids, a group of polyphenolic compounds widely distributed throughout the plant kingdom, are thought to contribute to the health benefits of diets rich in fruits and vegetables. The *in vivo *effects of several flavonoids (tea-containing catechins, soy isoflavones) have been reported in the literature.

The effect of tipi tea (*Petiveria alliacea*), a tea used as an antirheumatic medicine, on knee and hip OA was evaluated in a small 1-week crossover double-blind RCT against a placebo tea [119]. No significant differences as assessed in pain scores or functional assessment were found.

Regarding isoflavones, a 3-month double-blind RCT on knee OA failed to show that 40 g daily of soy protein, containing a total of 88 mg of soy isoflavones, was more efficient than a milk-based protein placebo in reducing symptoms as assessed by questionnaires on pain and quality of life [120]. Use of milk-based proteins as a placebo is confounding because milk could be effective in OA (see 'Milk and hyperimmune milk' section). Soy but not milk proteins increased serum levels of insulin-like growth factor-1, an anabolic factor for chondrocytes.

### *Boswellia serrata*

The gummy oleoresin from the bark of *B. serrata*, a tree from northwest India, is used for inflammatory diseases in Ayurvedic medicine. In an 8-week double-blind crossover RCT on knee OA, 333 mg of the gum three times a day was more efficient than placebo in reducing pain, loss of movement, and swelling scores [121]. In a 3-month double-blind RCT, 500 mg three times a day of a cocktail of *B. serrata *and turmeric (*Curcuma longa*) decreased categorical scales of joint pain, tenderness, and effusion [122]. According to the best-evidence synthesis, these RCTs provide no evidence of efficacy (Table 3).

*B. serrata *in combination with an extract from the root of *Withania somnifera*, the oleoresin of *C. longa*, and a zinc complex was tested in a 6-month double-blind crossover RCT [123]; 650 mg twice a day of this cocktail, called Articulin-F, was more efficient than placebo in reducing pain and disability scores. According to the best-evidence synthesis, this RCT indicates a lack of evidence of efficacy (Table 3).

### Cocktails of plant extracts

SKI306X is a cocktail of extracts prepared from three plants (dried roots from *Clematis mandshurica *and *Trichosantes kirilowii *and dried flower and stem from *Prunella vulgaris*) used for the treatment of inflammatory diseases in Far East Asia. It is clinically approved for the treatment of OA in Korea [124]. In a 4-week double-blind RCT on knee OA, 200, 400, and 600 mg three times a day were more efficient than placebo in reducing VAS of pain, with no significant difference between the three doses [125] (Table 4). In another 4-week double-blind RCT on knee OA, 200 mg three times a day was not less efficient than 100 mg of diclofenac (sustained release) once a day in reducing VAS of pain [126]. According to the best-evidence synthesis, these two high-quality RCTs provide moderate evidence for the efficacy of SKI306X in OA. Although generally well tolerated, three severe adverse events occurred in the SKI306X group (compared with 11 for diclofenac).

In rats, SKI306X did not cause significant gastric damage up to an oral dose of 2 g/kg and suppressed the diclofenac induced gastric damage [124]. In rabbits, 200 mg/kg per day reduced the OA-like histological changes in collagenase-injected knees [127]. *In vitro*, SKI306X and the *T. kirilowii *extract, but not the other two plant extracts, reduced the IL1α-induced GAG release [127]. Synergistic effects between the three extracts present in SKI306X are suspected because the proportion of *T. kirilowii *is insufficient to fully explain the product potency.

The effects of Gitadyl, an herbal formulation containing extracts from feverfew, American aspen, and milfoil, at a dose of 260 mg three times a day were compared with the effects of 400 mg of ibuprofen three times a day in a 42-day double-blind crossover RCT. Both treatments failed to significantly change pain or the patients' ability to walk as assessed by four point scales [128].

In a 4-week double-blind RCT on knee OA, 3 g three times a day of Duhuo Jisheng Wan, a traditional Chinese cocktail of 15 plants, improved the LFI and multiple VAS of pain and stiffness as efficiently as 25 mg of diclofenac three times a day [129] (Table 4). Duhuo Jisheng Wan had a slower onset of action than diclofenac but an equal rate of adverse events, an observation that questions its safety. According to the best-evidence synthesis, there is limited scientific evidence to support the efficacy of Duhuo Jisheng Wan (Table 3).

### Others

#### Methylsulfonylmethane

Methylsulfonylmethane (MSM) is the oxidised form of dimethyl sulfoxide. It is found in very low amounts in fruits, corn, tomatoes, tea, coffee, and milk. Two RCTs qualified to be evaluated in this systematic review (Table 4). In a 12-week double-blind placebo-controlled RCT on knee OA, 500 mg of MSM three times a day, used alone or in combination with 500 mg of glucosamine HCl three times a day, significantly improved a Likert scale of pain and LFI [130]. The combination of both ingredients was not more efficacious than each ingredient used alone. In a second 12-week double-blind placebo-controlled RCT on knee OA, 3 g of MSM given twice daily was more efficient than placebo in decreasing WOMAC pain and functional scores [131]. According to the best-evidence synthesis (Table 3), MSM provides moderate evidence of efficacy for knee OA.

#### Milk and hyperimmune milk

A cross-sectional epidemiological study suggested that the frequency of symptomatic knee OA was lower in milk consumers [132] but did not take into account body mass index, an important potential confounding factor [133]. Although no trial tested regular milk, one canine and two human double-blind RCTs tested hyperimmune milk. This milk is produced by cows that are immunised with intestinal bacteria antigens. It is enriched in high-molecular weight immunoglobulins (IgG) and is claimed to contain anti-inflammatory low-molecular weight components. A concentrated form of this milk was used in the RCTs. A 6-week human RCT failed to show that 355 ml a day of a fruit-flavoured beverage fortified with hyperimmune milk, vitamins B_12_, C, and E, and iron and zinc was more efficient than placebo in improving WOMAC [134]. In a 6-week three-arm RCT, 2 g twice a day of the milk preparation was not less efficient than 500 mg three times a day of glucosamine sulfate in improving WOMAC [135]. Unfortunately, a difference in symptomatic basal levels between treatment and placebo groups makes the placebo group of this RCT useless. Because none of these trials used regular milk as placebo, it is not known if hyperimmune milk has an effect different than regular milk. According to the best-evidence synthesis, there is a lack of scientific evidence of efficacy for hyperimmune milk.

In an 8-week RCT on dogs with musculoskeletal impairment, 1 g twice a day was more efficient than placebo in improving function as assessed by a newly developed questionnaire addressed to the pet owners [136]. Veterinarians' examination did not confirm these results. The absence of a precise diagnostic at enrolment and of any validation of the questionnaire limits the relevance of this study.

#### Collagen hydrolysate

Collagen hydrolysate is produced by enzymatic digestion of gelatin, which itself is produced by hydrolysis of collagen extracted from animal bones and skin.

In a 24-week multi-country double-blind placebo-controlled RCT on knee OA, 10 g/day did not improve the WOMAC index [137]. The dropout rate was high. Post hoc analysis suggested that the hydrolysate could be more efficient in severe OA. A 60-day double-blind crossover placebo-controlled RCT on knee and hip OA compared 10 g/day of collagen hydrolysate, gelatin, gelatin + glycin + CaHPO_4_*2H_2_O, or egg albumin [138]. The gelatin preparations were not significantly different from each other and were superior to egg albumin in reducing pain as assessed by a patient questionnaire. According to the best-evidence synthesis (Table 3), these two RCTs lack evidence of efficacy for collagen hydrolysate.

*In vitro*, type I or type II collagen hydrolysate dose-dependently increased type II collagen synthesis by chondrocytes, whereas native collagen and collagen-free hydrolysate did not [139]. The average molecular weight of collagen peptides in the hydrolysate ranges from 2 to 6 kDa. *Ex vivo *intestinal sac experiments suggested that peptides up to 15 kDa can be absorbed. In mice, a significant and long-lasting (>96 hours) accumulation of ^14^C-labeled collagen hydrolysate was observed in articular cartilage compared with ^14^C-labeled proline [140].

## Discussion

Fifty-three RCTs investigating the effects of functional ingredients in OA met the inclusion criteria for this systematic review. The functional ingredients tested in these RCTs were lipids (ASUs, n-3 PUFAs, lipid extracts from New Zealand green-lipped mussel, and cetyl myristoleate), vitamins and minerals (vitamins C, E, B_3_, and B_12_, boron, a cocktail of vitamins and selenium, and a cocktail of minerals), plant extracts (bromelain, *R. canina*, *H. procumbens*, *U. tomentosa *and *U. guianensis*, *Salix sp.*, ginger, turmerics, tipi tea, soy proteins, and *B. serrata*), a cocktail of plant extracts (SKI306X, Gitadyl, Duhua Jushing Wan, and Articulin-F), and a few other types of ingredients (methylsulfonlymethane, hyperimmune milk, and collagen hydrolysate). Eighteen of these functional ingredients had their efficacy supported by at least one RCT (Table 3).

To summarise the strength of scientific evidence behind a functional ingredient, we used a mathematically based best-evidence synthesis. This best-evidence synthesis allowed us to categorise the functional ingredients as having a limited, moderate, or good record of efficacy. According to this best-evidence synthesis (Table 3), good evidence exists for ASUs. Moderate evidence exists for methylsulfonylmethane and SKI306X, a cocktail of plant extracts. Limited evidence exists for the Chinese cocktail of plant extracts Duhuo Jisheng Wan, for cetyl myristoleate, for lipids from green-lipped mussels, and for plant extracts from *H. procumbens*. Limited evidence also exists for vitamins B_3 _and C and bromelain, but the small effects obtained, the high doses used, or the experimental design employed questions the clinical relevance and/or safety of these functional ingredients. The other interventions lacked scientific evidence either because of their rather poor design or because of contradicting available evidence. Among these interventions that lacked evidence of efficacy, vit E is unique: it is the only nutritional intervention whose lack of symptom-modifying and structure-modifying effects in knee OA is reported in high-quality RCTs. Despite the fact that our best-evidence synthesis considers each functional ingredient as a single entity, the evidence of efficacy and the safety record of plant extracts should be considered to be product-specific given that the composition of an extract from a same plant can vary widely between manufacturers.

All 18 functional ingredients evaluated in Table 3 were tested under a nutraceutical/dietary supplement form in the RCTs, except for hyperimmune milk incorporated in a functional drink. Depending on the regulatory laws of each country, these functional ingredients are sold as drugs, nutraceuticals (dietary supplements), or functional foods in association with health claims of variable strength. Although most ingredients are sold mostly as nutraceuticals today, some such as SKI306X and ASUs require a prescription and are sold as drugs, at least in some countries (Korea for SKI306X and several European countries for ASU). Similarly, the vitamins and some of the lipids reviewed here are sold mostly as nutraceuticals but can also be incorporated in functional foods (up to a country-specific defined maximal dose) because they have GRAS (generally recognised as safe) status. Regarding collagen hydrolysate specifically, its GRAS status and its advertised therapeutic dose (10 g) make it more practical to be used in a functional food than in a nutraceutical. Ideally, the efficacy of a functional food should be directly evaluated in an RCT (by providing to the enrolled patients the final commercial product) because the incorporation of a functional ingredient into a complex food matrix could potentially modify its efficacy, either by increasing or on the contrary by decreasing its bioavailability.

## Conclusion

In summary, this review demonstrates that nutrition can improve the symptoms of declared OA. However, the role of nutrition in slowing down progression of the disease remains to be seen. The very few RCTs, which used structure-modifying variables as primary endpoints, were unable to demonstrate a benefit, but the area deserves further investigation. As a whole, nutritional research in OA is only in its infancy. Only a few ingredients have been tested, and research remains based mainly on a pharmacological type of approach (one molecule/one target) rather than on a nutritional, more holistic type of approach (multiple ingredients/multiple targets). The full potency of nutrition for patients with declared OA thus remains to be evaluated. In parallel, and except for a few longitudinal epidemiological studies on vitamins, no study has evaluated the value of nutrition in the prevention of OA. Although these studies are of utmost importance, the size, the duration, and hence the prohibitive cost of such studies, particularly in the form of human intervention trials, keep them beyond our reach for the time being. This situation will probably persist at least until we considerably improve our prognostic tools to detect those 'healthy' subjects at high risk of developing OA in their near future.

## Appendix

### Comparative discussion on the value of the Jadad and OA scores to evaluate the quality of clinical trials on OA

To evaluate the quality of the RCTs, we used two scores: the previously validated Jadad score [21], which can be used to score any type of clinical trials, and a new OA score designed especially for this study and tailor-made for OA clinical trials. According to these two scoring systems, the quality of the RCTs was highly heterogeneous. Based on the OA score, the quality of the trials ranged from 33% to 100% (with a mean of 65 and a median of 67) (Table 2). Based on the Jadad score, the quality of the trials ranged from 20% (that is, a score of 1 out of a possible maximum of 5) to 100% (that is, a score of 5) with an average and median score of 80%. Conceptual differences in design exist between the two scoring systems. The Jadad score evaluates only the randomisation method, the double-blinding method, and the report of dropouts, whereas the OA score is more comprehensive. Between the two authors, the reproducibilities of the two instruments were similar (approximately 92%–93%). Although the two scores were overall quite consistent with each other, divergence sometimes emerged between them due to their different designs ([47,72,85,110,115,121,135]; Table 3). Due to its higher complexity, the OA score was more powerful than the Jadad score in discriminating the quality of the trials. Indeed, several trials, despite a maximal Jadad score and 1 point allocated to the three criteria of the OA score evaluated in the Jadad score (that is, criteria numbers 4, 6, and 11), ended up with very different OA scores, ranging from 0.42 to 1 (compare, for example, the scores of [26] and [47] in Table 3). Based on this observation and in agreement with published guidelines recommending the development and use of disease-specific scoring systems for systematic reviews [141], the OA score seems more accurate than the Jadad score in evaluating the quality of RCTs on OA.

## Abbreviations

AGE = advanced glycation endproduct; ASU = avocado soybean unsaponifiable; COX = cyclo-oxygenase; CRP = C-reactive protein; GAG = glycosaminoglycan; GRAS = generally recognised as safe; IL = interleukin; LFI = Lequesne functional index; LOX = lipo-oxygenase; LPS = lipopolysaccharide; MMP = matrix metalloproteinase; MSM = methylsulfonylmethane; NF = nuclear factor; NO = nitric oxide; NSAID = nonsteroidal anti-inflammatory drug; OA = osteoarthritis; PGE2 = prostaglandin E2; PUFA = poly-unsaturated fatty acid; RCT = randomised clinical trial; RDA = recommended daily allowance; ROS = reactive oxygen species; SAMe = S-adenosyl-L-methionine; TNF = tumour necrosis factor; VAS = visual analog scale; vit = vitamin; WOMAC = Western Ontario and McMaster universities [index].

## Competing interests

Both authors are employees of Nestec S.A.

## Authors' contributions

LGA conceived the review, collected and read the quoted references, scored the clinical trials, drafted Tables 1, 2, 3, and wrote the manuscript. WSSC scored the clinical trials, drafted Table 4, and revised the final version of the manuscript. Both authors read and approved the final manuscript.

## Figures and Tables

**Table 1:** Numbers of papers remaining after each stage of the selection process of the systematic review

Raw hits from all sources	2,026
Number of studies reviewed for inclusion criteria after reading the title	121
Number of studies excluded because: administration was not oral of pharmacological interventions they did not report the result of a clinical trial clinical trials were not randomised results already reported in another paper (duplicate reports)	-23 -13 -30 -2 -2
Number of RCTs found during the reviewing process by serendipity and added to the review	+2
Number of RCTs matching inclusion criteria and reviewed here	53
Number of negative RCTs that concerned nutritional intervention for which no positive RCT was found	-11
Number of RCTs the quality of which was scored (Table 3)	42

**Table 2:** Criteria used for the assessment of the methodological quality of human clinical trials

Item	Criterion
Study population	
1.	Patients with radiographically confirmed osteoarthritis or selected according to American College of Rheumatology guidelines
2.	Age, gender, and body mass index reported and not statistically different between groups
3.	Efficacy assessed on a single anatomical joint (for example, knee)
Trial design	
4.	Randomisation
5.	Placebo-controlled study
6.	Double-blind study
7.	Duration of at least 3 months
8.	Selection of a single primary endpoint before beginning of trial
9.	Sample size based on power calculation
Analysis and data presentation	
10.	Data analysed according to the intention-to-treat principle
11.	Reported dropout rate not more than 25%
12.	Report of adverse effects

**Table 3:** Ingredients, with the scores of the trials^a^, displayed by decreasing order of strength of evidence

**Nutritional intervention **Trial (Author's name/year) [reference number]	Was treatment efficient?	OA score													Jadad score of the RCT	Global score of the functional ingredients
				
		Criterion number												Totalscore of the RCT		
					
		1	2	3	4	5	6	7	8	9	10	11	12			
**Avocado soybean unsaponifiables**																**1.58**
Blotman 1997 [24]	Yes	1	1	0	1	1	1	1	1	1	1	1	1	0.92	5	
Maheu 1998 [26]	Yes	1	1	1	1	1	1	1	1	1	1	1	1	1	5	
Appelboom 2001 [25]	Yes	1	0	1	1	1	1	1	1	0	1	1	1	0.83	3	
Lequesne 2002 [27]^b^	No	1	1	1	1	1	1	1	1	1	1	0	1	-0.92	5	**-0.92**
**Methylsulfonylmethane**																**1.21**
Usha 2004 [130]	Yes	1	1	1	1	1	1	1	0	1	1	1	1	0.92	5	
Kim 2006 [131]	Yes	1	0	1	1	1	1	1	0	1	1	1	1	0.83	5	
**SKI306X**																**1.12**
Jung 2001 [125]	Yes	1	1	1	1	1	1	0	1	1	1	0	1	0.83	4	
Jung 2004 [126]	Yes	1	0	1	1	0	1	0	1	1	1	1	1	0.75	5	
**Vitamin B_3_**																**0.75**
Jonas 1996 [79]	Yes	1	1	0	1	1	1	1	0	1	0	1	1	0.75	5	
**Vitamin C**																**0.75**
Jensen 2003 [58]	Yes	1	0	0	1	1	1	0	1	1	1	1	1	0.75	5	
**Duhuo Jisheng Wan**																**0.67**
Teekachunhatean 2004 [129]	Yes	1	1	1	1	0	1	0	0	0	1	1	1	0.67	3	
**Lipids from *Perna canaliculus***																**0.58**
Gibson 1980 [46]	Yes	1	0	0	1	1	1	1	0	0	0	1	1	0.58	4	
Audeval 1986 [45]	Yes	1	0	1	1	1	1	1	1	0	0	1	1	0.75	4	
Gibson 1998 [47]	Yes	1	0	0	1	0	1	1	0	0	0	1	0	0.42	5	
**Cetyl myristoleate**																**0.58**
Hesslink 2002 [50]	Yes	1	1	1	1	1	1	0	0	0	0	1	0	0.58	3	
***Harpagophytum procumbens***																**0.54**
Lecomte 1992 [99]	Yes	0	0	0	1	1	1	0	0	0	0	0	1	0.33	3	
Chantre 2000 [100]	Yes	1	0	0	1	0	1	1	1	1	1	1	1	0.75	5	
**Bromelain**																**0.53**
Singer 1996 [92]	Yes	1	0	1	1	0	1	0	0	0	1	1	1	0.58	4	
Klein 2000 [91]	Yes	1	0	1	1	0	1	0	1	0	1	1	0	0.58	4	
Singer 2001 [142]	Yes	1	0	1	1	0	1	0	0	0	1	1	1	0.58	4	
Tilwe 2001 [89]	Yes	1	0	1	1	0	0	0	0	0	1	0	0	0.33	2	
Akhtar 2004 [90]	Yes	1	1	1	1	0	1	0	0	0	1	0	1	0.58	4	
**Boron**																**0.50**
Newnham 1994 [85]	Yes	1	0	0	1	1	1	0	0	0	0	1	1	0.50	4	
***Uncaria *guianensis**																**0.50**
Piscoya 2001 [106]	Yes	1	0	1	1	1	1	0	0	0	0	0	1	0.50	3	
***Boswellia serrata***																**0.48**
Kulkarni 1991 [123]^c^	Yes	1	0	0	1	1	1	1	0	0	0	0	1	0.50	3	
Badria 2003 [122]	Yes	0	0	1	1	1	1	1	0	0	0	0	0	0.42	3	
Kimmatkar 2003 [121]	Yes	1	0	1	1	1	1	0	0	0	0	1	0	0.50	5	
**Ginger**																**0.42**
Bliddal 2000 [115]	No	1	0	0	1	1	1	0	0	1	0	1	1	-0.58	5	
Altman 2001 [113]	Yes	1	1	1	1	1	0	0	1	0	1	1	1	0.75	3	
Wigler 2003 [112]	Yes	1	0	1	1	1	1	1	0	0	1	0	1	0.67	5	
**Vitamin E**																**0.17**
Machtey 1978 [71]	Yes	1	0	0	1	1	0	0	1	0	0	1	1	0.50	1	
Blankenhorn 1986 [72]	Yes	1	0	0	1	1	1	0	0	0	0	1	1	0.50	4	
Scherak 1990 [73]	Yes	1	0	0	1	0	1	0	0	0	0	1	1	0.42	3	
Brand 2001 [74]	No	1	0	1	1	1	1	1	1	1	1	1	1	-0.92	5	
Wluka 2002 [75]^b^	No	1	1	1	1	1	1	1	1	1	1	1	0	-0.92	5	**-0.92**
**'Hyperimmune' milk**																**-0.09**
Colker 2002 [134]	No	0	1	1	1	1	1	0	1	0	0	1	1	-0.67	4	
Zenk 2002 [135]	Yes	0	1	0	1	0	1	0	1	0	0	1	1	0.50	5	
**Collagen hydrolysate**																**-0.17**
Adam 1991 [138]	Yes	1	0	0	1	1	1	0	0	0	0	0	0	0.33	3	
Moskowitz 2000 [137]	No	1	0	1	1	1	1	1	0	0	1	0	1	-0.67	3	
***Salix sp*.**																**-0.25**
Mills 1996 [110]^d^	No	0	0	0	1	1	1	0	0	0	0	1	1	-0.42	5	
Schmid 2001 [108]	Yes	1	1	0	1	1	1	0	1	0	1	1	1	0.75	5	
Biegert 2004 [109]	No	1	1	0	1	1	1	0	1	1	1	1	1	-0.83	5	

Each item of the osteoarthritis (OA) score was given 1 point when it met the specified criterion listed in Table 2. If it did not meet the criterion or was not described at all, a score of 0 was assigned. For each trial, the sum of the individual scores was expressed as a percentage to give a relative total quality score. ^a^To be included in this table, any functional ingredient had to have its efficacy supported at least by one trial. This was considered to be the case when a statistical difference in the primary endpoint of a clinical trial was observed or, in the absence of a defined primary endpoint, when statistical differences were observed in several of the reported endpoints. ^b^Randomised human clinical trial (RCT) evaluating the structure-modifying effects of the functional ingredients. ^c^Cocktail of three plant extracts and zinc complex.^d^Cocktail of five plant extracts among which one extract from *Salix sp*.

**Table 4:** Summary of trials on ingredients having at least a limited evidence of efficacy

**Lead author and date [Reference]**	**Inclusion criteria**	**Duration of intervention, study design, sample size and treatment (dosage)**	**Sample size and dropout rate (percentage) at the end of treatment**	ITT results at the end of treatment (baseline and final values or percentage change, intergroup *p *value)
**ASUs**				

Blotman 1997 [24]	Knee and hip OA Mean age = 64.1 years Mean wt = 70.2 kg Mean ht = 166 cm F/M: 108/55	3 months Parallel study (*n *= 164) 1. Placebo (*n *= 83) 2. ASU (*n *= 81) (300 mg × 1/day)	Placebo (*n *= 76) ASU (*n *= 77) Dropout = 6.7%	Number of patients who resumed NSAID intake Placebo (*n *= 53) (69.7%) ASU (*n *= 33) (43.4%) *p *< 0.001
Maheu 1998 [26]	Knee and hip OA Mean age = 64.1 years Mean BMI = 26.8 F/M: 118/46	6 months Parallel study (*n *= 164) 1. Placebo (*n *= 79) 2. ASU (*n *= 84) (300 mg × 1/day)	Placebo (*n *= 69) ASU (*n *= 75) Dropout = 12%	LFI score: Placebo (9.3 to 9.9, +6%) ASU (9.7 to 6.8, -30%) *p *< 0.001
Appelboom 2001 [25]	Knee OA Mean age = 65 years Mean wt = 76.5 kg Mean ht = 164 cm F/M: 205/55	3 months Parallel study (*n *= 260) 1. diclofenac (*n *= 88) 2. ASU (*n *= 86) (300 mg × 1/day) 3. ASU (*n *= 86) (600 mg × 1/day)	Placebo (*n *= 76) ASU 300 mg (*n *= 74) ASU 600 mg (*n *= 75) Dropout = 13.5%	Intake of NSAID and analgesics intake (mg/diclofenac per day) Placebo (130 to 81, -38%) ASU 300 mg (133.8 to 45.2, -66%) ASU 600 mg (123.7 to 52.5, -58%) *p *< 0.01 for each ASU group vs. placebo ASU 300 vs. ASU 600: NS
Lequesne 2002 [27]	Hip OA Mean age = 63.2 years Mean wt = 70.5 kg Mean ht = 165 cm F/M: 61/102	2 years Parallel study (*n *= 163) 1. Placebo (*n *= 78) 2. ASU (300 mg × 1/day) (*n *= 85)	Placebo (*n *= 45) ASU (*n *= 51) Dropout = 41.1%	Joint space width mm: Placebo: 2.50 to 1.90, -24% ASU: 2.35 to 1.87, -20% NS between groups

**MSM**				

Usha 2004 [130]	Knee OA Mean age = 51 years Mean wt = 66 kg Mean ht = 160.5 cm F/M: 76/42	12 weeks Parallel study Double dummy (*n *= 118) 1. Placebo (*n *= 28) 2. Glu (500 mg × 3/day) (*n *= 30) 3. MSM (500 mg × 3/day) (*n *= 30) 4. Glu (500 mg × 3/day) + MSM (500 mg × 3/day) (*n *= 30)	Placebo (*n *= 24) Glu (*n *= 27) MSM (*n *= 27) Glu + MSM (*n *= 28) Dropout = 10.2%	Likert scale pain index (0 to 3) Placebo (1.57 to 1.16, -26%) Glu (1.74 to 0.65, -63%) *p *< 0.001 vs. placebo MSM (1.53 to 0.74, -52%) *p *not reported Glu + MSM (1.7 to 0.36, -79%) *p *< 0.05 vs. Glu and MSM alone LFI Placebo: NS decrease Glu: 13 to 8.85, -32% MSM: 12.48 to 8.48, -32% Glu + MSM: 13 to 8.65, -33% *p *between groups not reported
Kim 2006 [131]	Knee OA Mean age = 56 years F/M: 25/15	12 weeks Parallel study (*n *= 50) 1. MSM (*n *= 25) (6 g/day) 2. Placebo (*n *= 25)	Placebo (*n *= 19) MSM (*n *= 21) Dropout = 20%	WOMAC pain: Placebo (55.1 to 47.9, -13.2%) MSM (58 to 43.4, -25%) *p *= 0.041 WOMAC stiffness Placebo (55.2 to 48.7, -12%) MSM (51.2 to 41.1, -19.7%) *p *= 0.32 WOMAC physical function Placebo (52.9 to 44.1, -16.6%) MSM (51.5 to 35.8, -30.4%) *p *= 0.045 WOMAC total Placebo (54.4 to 46.9, -13.8%) MSM (53.6 to 40.1, -25%) *p *= 0.054

**SKI306X**				

Jung 2001 [125]	Knee OA Mean age = 58 years Mean wt = 62.2 kg Mean ht = 157.1 cm F/M: 84/9	4 weeks Parallel study (*n *= 96) 1. Placebo (*n *= 24) 2. SKI (200 mg × 3/day) (*n *= 24) 3. SKI (400 mg × 3/day) (*n *= 24) 4. SKI (600 mg × 3/day) (*n *= 24)	Placebo (*n *= 23) SKI (200 mg) (*n *= 24) SKI (400 mg) (*n *= 23) SKI (600 mg) (*n *= 23) Dropout = 3%	VAS of pain (only absolute change reported) Placebo: -7.5 mm 200 mg: -23.6 mm 400 mg: -22.0 mm 600 mg: -29.8 mm *p *< 0.001 for each SKI306X group vs. placebo
Jung 2004 [126]	Knee OA Mean age = 60 years F/M: 231/18	4 weeks Parallel study Double dummy (*n *= 249) 1. diclofenac (*n *= 124) 2. SKI (200 mg × 3/day) (*n *= 125)	Diclofenac (*n *= 109) SKI (*n *= 105) Dropout = 14.1%	VAS of pain (only absolute change reported) Diclofenac -15.49 mm SKI -14.18 mm NS between groups

**Vitamin B_3_**				

Jonas 1996 [79]	OA of at least two joints Mean age = 65 years Mean wt = 163 kg F/M 38/22 (PP)	12 weeks (N = 72) 1. Placebo 2. Vit B_3 _(*n *= 500 mg/day × 6/day)	Placebo (*n *= 29) Vit B_3 _(*n *= 31) Dropout = 17%	Global AIMS score (only change reported) Placebo: +2.7, -10% Vit B_3_: -5.9, +29% *p *= 0.036 NSAIDs intake (pill/month) (only change reported) Placebo: +0.25 Vit B_3_: -6.7, -13% *p *= 0.014 VAS pain (only change reported) Placebo: +1 mm Vit B_3_: +8.2 mm NS between groups

**Vitamin C**				

Jensen 2003 [58]	OA hip and/or knee Mean age = 63 years All female	14 days Crossover study 7 days washout (*n *= 136) 1. Placebo 2. Calcium ascorbate (Vit C) (1,000 mg × 2/day)	Placebo (*n *= 71) Vit C (*n *= 62) Dropout = 2.2%	VAS pain: Average difference between gps before and after crossover: 4.6 mm (starting levels 45–50 mm) *p *= 0.0078

**Duhuo Jisheng Wan**				

Teekachunhatean 2004 [129]	Knee OA Mean age = 62.5 years Mean BMI = 26 F/M: 159/41	4 weeks Parallel study Double dummy (*n *= 200) 1. Diclofenac (25 mg × 3/day) (*n *= 100) 2. DJW (3 g × 3/day) (*n *= 100)	Diclofenac (*n *= 94) DJW (*n *= 94) Dropout = 6%	VAS total pain mm (sum of 5 VAS) DJW (269 to 70, -73.93%) Diclofenac (267 to 58, -78.15%) VAS total stiffness cm (sum of 3 VASs) DJW (122 to 32, -73.81%), Diclofenac (129 to 32, -75.30%) LFI DJW (14.20 to 8.92, -37.18%) Diclofenac (14.80 to 8.64, -41.62%) NS between groups for all

**Cetyl myristoleate**				

Hesslink 2002 [50]	Knee OA Mean age = 56.8 years Mean wt = 76.4 kg Mean ht = 164.7 cm F/M: 39/25	68 days Single-blind parallel study (*n *= 66) 1. Placebo: (soy lecithin 500 mg) 2. Cetyl myristoleate (350 mg, 50 mg soy, 75 mg fish oil)	Placebo (*n *= 31) Cetyl myristoleate (*n *= 33) Dropout 3%	Knee flexion Cetyl myristoleate: +10.1 degree Placebo: +1.1 degree *p *< 0.001.

**Lipids from *Perna canaliculus***				

Gibson 1980 [46]	OA hip and knee Mean age = 55 years F/M: 22/8	3 months Parallel study (*n *= 38) 1. Lipid extract (210 mg/day) (*n *= 22) 2. Mussel powder (1,150 mg/day) (*n *= 16)	Dropout = 13%	VAS pain: Placebo: 13% improved Mussel powder 40% improved
Audeval 1986 [45]	Knee OA Mean age = 66 years F/M: 37/16	6 months Parallel study (*n *= 53) 1. Placebo 2. Mussel powder (dose not stated)	Dropout = 0%	VAS pain mm Placebo (59 to 68, +15%) Mussel powder (54 to 27, -50%) *p *< 0.001
Gibson 1998 [47]	OA hip and knee Mean age = 69 years F/M: 37/1	3 months Parallel study (*n *= 30) 1. Lipid extract (210 mg/day) (*n *= 15) 2. Mussel powder (1,150 mg/day) (*n *= 15)	Lipid (*n *= 13) Mussel powder (*n *= 13) Dropout = 13%	VAS pain (absolute values not reported) Difference between groups not reported *p *< 0.05 vs. baseline for both groups

***Harpagophytum procumbens***				

Lecomte 1992 [99]	OA spine and knee 55 to 75 years old F/M: 50/39	2 months Parallel study (*n *= 89) 1. Placebo (*n *= 44) 2. *H. procumbens *(670 mg × 3/day) (*n *= 45)	Not reported	VAS pain mm Placebo (68 to 50,-26%) *H. procumbens *(73 to 45, -38%) *p *= 0.012
Chantre 2000 [100]	OA hip and knee Mean age = 62 years Mean wt = 75 kg F/M: 77/55	4 months Parallel study Double dummy (*n *= 112) 1. Diacerhein (50 mg × 2/day) (*n *= 60) 2. *H. procumbens *(Harpado) (435 mg × 6) (*n *= 62)	Diacerhein (*n *= 42) *H. Procumbens *(*n *= 50) Dropout = 27%	VAS pain cm Diacerhein (62 to 36, -42%), *H. procumbens *(64 to 31, -51%) NS between groups

**Bromelain**				

Singer 1996 [92]	Knee OA Mean age = 53 years F/M 37/43	28 days Parallel study Double dummy (*n *= 63) 1. Diclofenac (50 mg × 2/day) (*n *= 40) 2. Phlogenzym (Bromelain 90 mg × 2/day) (*n *= 40)	Diclofenac (*n *= 36) Phlogenzym (*n *= 32) Dropout rate = 15%	Morning pain (score 1–5) Diclofenac (2.5 to 1.2, -52%) Phlogenzym (2.3 to 1.4, -39%) NS between groups Pain walking (score 1–5) Diclofenac (3.1 to 1.4, -55%) Phlogenzym (2.9 to 1.7, -41%) NS between groups
Singer 2001 [142]	Knee OA 19–75 years	21 days Parallel study Double dummy (*n *= 63) 1. Diclofenac (50 mg × 2/day) (*n *= 32) 2. Phlogenzym (Bromelain 90 mg × 2/day) (*n *= 31)	Dropout rate = 0%	VAS pain at rest mm Diclofenac: 31 to 14, -54% Phlogenzym: 35 to 15, -58% NS between groups VAS pain on movement cm Diclofenac: 54 to 27, -49% Phlogenzym: 60 to 30, -56% NS between groups LFI: Diclofenac: 15.81 to 12.77, -19% Phlogenzym 15.48 to 9.81, -37% *p *= 0.0165
Klein 2000 [91]	Knee OA Mean age = 52 years F/M: 37/36	3 weeks Parallel study Double dummy (*n *= 73) 1. Diclofenac (50 mg × 2/day) (*n *= 37) 2. Phlogenzym (Bromelain 90 mg × 2/day) (*n *= 36)	Diclofenac (*n *= 34) Phlogenzym (*n *= 35) Dropout = 5.5%	LFI: Diclofenac: 14.04 to 3.50, -75% Bromelain 13.56 to 3.10, -77% NS between groups
Tilwe 2001 [89]	Knee OA Mean age = 57 years F/M: 31/19	3 weeks Parallel study Single-blind study (*n *= 50) 1. Phlogenzym (45 mg bromelain × 2/day) (*n *= 25) 2. Diclofenac (50 mg × 2/day) (*n *= 25)	Not reported	Joint tenderness (4-pt scores) Diclofenac 1.44 to 1.16, -19.4% bromelain 1.64 to 0.80, -51.2%, *p *< 0.05 Pain at rest (4-pt scores) Diclofenac 1.24 to 0.92, -25.8% bromelain 1.12 to 0.64, -42.9%, NS difference between groups Pain on movement (4-pt scores) Diclofenac 2.04 to 1.32, -35.3% bromelain 1.92 to 1.16, -39.6%, NS difference between groups
Akhtar 2004 [90]	Knee OA Mean age = 57 years Mean wt = 76 kg Mean ht = 163 cm F/M: 70/28	6 weeks Parallel study Double dummy (*n *= 98) 1. Phylogenzym (90 mg bromelain × 3/day) (*n *= 46) 2. Diclofenac (50 mg × 2/day) (*n *= 52)	Diclofenac (*n *= 42) Phylogenzym (*n *= 36) Dropout = 20%	LFI Diclofenac 12.5 to 9.4, -23.6%, Phlogenzym 13.0 to 9.4, -26.3% NS difference between groups

ASU = avocado soybean unsaponifiable; BMI = body mass index; DJW = Duhuo Jisheng Wan; F = female; Glu = glucosamine; ht = height; ITT = intention-to-treat; LFI = Lequesne functional index; M = male; MSM = methylsulfonyl methane; N = total sample size; NS = not significant; NSAID = nonsteroidal anti-inflammatory drug; OA = osteoarthritis; PP = per protocol; SKI = SKI 306X; VAS = visual analog scale; Vit = vitamin; WOMAC = Western Ontario and McMaster universities [index]; wt = weight.
